# Development of a classification model for *Cynanchum wilfordii* and *Cynanchum auriculatum* using convolutional neural network and local interpretable model-agnostic explanation technology

**DOI:** 10.3389/fpls.2023.1169709

**Published:** 2023-06-02

**Authors:** Dae-Hyun Jung, Ho-Youn Kim, Jae Hee Won, Soo Hyun Park

**Affiliations:** ^1^ Department of Smart Farm Science, Kyung Hee University, Yongin-si, Republic of Korea; ^2^ Smart Farm Research Center, Korea Institute of Science and Technology (KIST), Gangneung-si, Republic of Korea; ^3^ Division of Bio-Medical Science and Technology, Korea Institute of Science and Technology (KIST) School, University of Science and Technology (UST), Daejeon, Republic of Korea; ^4^ Quality Certification Center, National Development Institute of Korean Medicine (NIKOM), Daegu, Republic of Korea

**Keywords:** explainable artificial intelligence, image classification, Incpetion-ResNet, sensory evaluation analysis, image classification algorithm

## Abstract

*Cynanchum wilfordii* is a perennial tuberous root in the Asclepiadaceae family that has long been used medicinally. Although *C. wilfordii* is distinct in origin and content from *Cynancum auriculatum*, a genus of the same species, it is difficult for the public to recognize because the ripe fruit and root are remarkably similar. In this study, images were collected to categorize *C. wilfordii* and *C. auriculatum*, which were then processed and input into a deep-learning classification model to corroborate the results. By obtaining 200 photographs of each of the two cross sections of each medicinal material, approximately 800 images were employed, and approximately 3200 images were used to construct a deep-learning classification model *via* image augmentation. For the classification, the structures of Inception-ResNet and VGGnet-19 among convolutional neural network (CNN) models were used, with Inception-ResNet outperforming VGGnet-19 in terms of performance and learning speed. The validation set confirmed a strong classification performance of approximately 0.862. Furthermore, explanatory properties were added to the deep-learning model using local interpretable model-agnostic explanation (LIME), and the suitability of the LIME domain was assessed using cross-validation in both situations. Thus, artificial intelligence may be used as an auxiliary metric in the sensory evaluation of medicinal materials in future, owing to its explanatory ability.

## 1 Introduction

Northeast Asian countries have long employed medical plants, such as herbal medicines, in the private sector to treat chronic ailments, and the industry is constantly developing. Functional compounds extracted from medicinal plants, in particular, are being employed as components of health functional meals, and efficacy studies as pharmaceutical substances are being actively done. Thus, the value of their use is quite high (Jiang et al., 2011). As the need for medical plants grows, so do related industrial achievements. However, the requirement for periodic standardization and stability difficulties before production, distribution, and sales management is growing (Han et al., 2016). This is a fundamental issue to address in maintaining the equivalence of efficacy and quality control of pharmaceuticals employing accurate medicinal ingredients, and ensuring such quality is a critical aspect in standardizing and modernizing oriental medicine products for entry into the global market.

*Cynanchum wilfordii* is a perennial tuberous plant of the *Asclepiadaceae* family that grows in Korea, China, and Japan. It has traditionally been used for nutrition, tonic, blood, and renal health. Among the components, gagaminine has been shown to inhibit hepatic aldehyde oxidase, and cynandione A has been shown to inhibit nerve cell damage (Ryuk et al., 2014). Comparing the chemical composition of the two herbs, Caudatin-2,6-dideoxy-3-O-methy- β-D-cymaropyranoside and caudatin isolated from C. auriculatum Royle ex Wight showed high antitumor activity against SMMC-7721 cells and inhibited H22 tumor growth *in vivo* (Peng et al., 2008). Meanwhile, C. wilfordii roots yielded nine new C21 steroidal glycosides, cynawilfosides A–I, and 12 known compounds(Li et al., 2016). C. *wilfordii* is not related to *C. auriculatum* in origin or content. However, recognizing it is challenging for the public owing to the similarity between the ripe fruit and seed. *C. auriculatum*, in particular, was designated as a toxic plant. However, two separate raw materials are blended and placed into the market before ensuring raw material stability, which might lead to consumer distrust of the mixed product (Ryu et al., 2018). It is particularly challenging to detect whether or not it is blended since it is processed in the form of powder or extract.

The traditional method of identifying medicines through morphological characteristics such as appearance, color, taste, and smell is widely used in the field because it has the advantage of being simple and direct confirmation. However, its fundamental flaw is dependent on the discriminator’s expertise and subjectivity (Jiang et al., 2011; Ryu et al., 2018). Various methods have been developed to compensate for these disadvantages. Internal morphology identification using microscopy and anatomical techniques, physicochemical identification that analyzes and compares components, and gene identification through unique genetic information search through genome analysis (Kim et al., 2013). A method using DNA markers, which are not affected by external factors, has recently been actively studied for major crops to identify plant species accurately and quickly. Unlike morphological characteristics such as the shape and size of plants, the method using DNA markers can distinguish plant species without being affected by the external environment. Furthermore, no limit exists on the number of markers that can be used. Therefore, an accurate discrimination is possible (Sato-Masumoto et al., 2017). However, it is necessary to secure the DNA nucleotide sequence of all medicinal materials, and there is a limit to immediately distinguishing raw materials in the field.

The spread of smartphones and the development of deep-learning-based image recognition technology have laid the foundation for easily recognizing various objects and acquiring information through cameras (Sun and Qian, 2016; Afonso et al., 2020). The field of plant recognition is a good use case that can apply the advancement of these technologies, and various technological studies are being conducted. Furthermore, services based on this have become an environment that is easily accessible through mobile devices (Grinblat et al., 2016; Saleem et al., 2019; Xi and Panoutsos, 2020; Jung et al., 2022). The massive amount of ImageNet data has had a profound impact on the development of machine learning technology in the field of image recognition vision. In particular, deep-learning technology, which has developed existing neural network technology, has shown rapid performance improvement. It has been applied to almost all fields that require artificial intelligence (AI), such as vision and natural language processing, and research is still being actively conducted. Plant recognition technology mainly utilizes convolutional neural networks (CNN) among deep-learning technologies. Recognizing the type of plant captured by the camera is one of the primary purposes of plant recognition. Because of this, plants become the main subject, and usability is sufficiently increased by classification alone. Dyrmann et al. (2016) developed a CNN model with 86.2% classification accuracy for 22 weed and crop species by learning 10,413 images, including 22 weed and crop species. Lin et al. (2019) applied a CNN-based deep-learning model to classify Powdery Mildew in cucumber and reported a classification accuracy of about 96.08%.

Deep-learning technology is widely applied in the plant recognition field with the development of deep-learning technology. However, there are not many cases of collecting and analyzing large amounts of images of medicinal plants. In particular, the most important point in applying image-based deep-learning classification technology is to enhance understanding of which patterns and parts of the sample were identified to predict the results for plants such as Cynanchum wilfordii, which are difficult to distinguish due to the large number of similar varieties with the naked eye.

Because these medicinal samples are easily jumbled during the processing and distribution stages, they always necessitate the services of a sensory evaluation expert (Chong and Cros, 2004). Numerous images are required in this sensory evaluation area to translate morphological features into data, and it is critical to locate the feature locations. However, due to the black-box nature of neural network-based modeling, it is nearly hard to interpret, making model prediction challenging to understand. Sun and Qian (2016) used CNN technology for herbal medicine image recognition to classify 95 categories of medicinal herbs from 5523 images. As a result, it reported 71% recognition accuracy and 53% retrieval accuracy on average when learning. This demonstrates that there is still a tremendous possibility for improvement if we contribute to learning by spotting similarities among therapeutic ingredients.

Consequently, Explainable Artificial Intelligence (XAI) technology, which can provide AI interpretation, is gaining popularity. By building more explainable models while maintaining high levels of performance, XAI enables analysts to understand and trust AI (Apostolopoulos et al., 2022; Başaran, 2022). The goal of XAI was defined in three ways: The first is to reduce the model’s complexity, the second is to improve the predictability of model predictions, and the third is to employ AI models for decision-making. One of the existing detection models, a rule-based model, was proposed in early XAI as a model that conducts detection based on rules that analysts can understand. Zhen et al. suggested a CNN learning structure with an interpretability-oriented layer in the form of fuzzy logic-based rules. The LRP (Layer-wise Relevance BackPropagation) model was proposed as a method of backtracking the NN (Neural Network) model’s outcomes and calculating the contribution of input data to individual features (Bach et al., 2015). Local Interpretable Model-Agnostic Explanations (LIME) is an explanation technique for local models that focuses on training local surrogate models to explain prediction outcomes (Ribeiro et al., 2016; Tulio Ribeiro et al., 2016). LIME is frequently used for image-based active site prediction. It looks for ‘superpixels’ with the highest expressiveness for binary vectors or class outputs that signal the presence or absence of continuous pathways. Several research cases have been published, particularly those that learn characteristics and enhance confidence in CNN algorithms.

CNN models capable of distinguishing *C. wilfordii* and *C. auriculatum* are used in this study to verify the viability of image-based categorization among herbal medications. Furthermore, this study aims to contribute to the advancement of deep-learning recognition models by employing LIME, which can express morphological features using explainable AI technology.

## 2 Materials and methods

### 2.1 Acquisition of image data and preprocessing method

**Figure 1:**
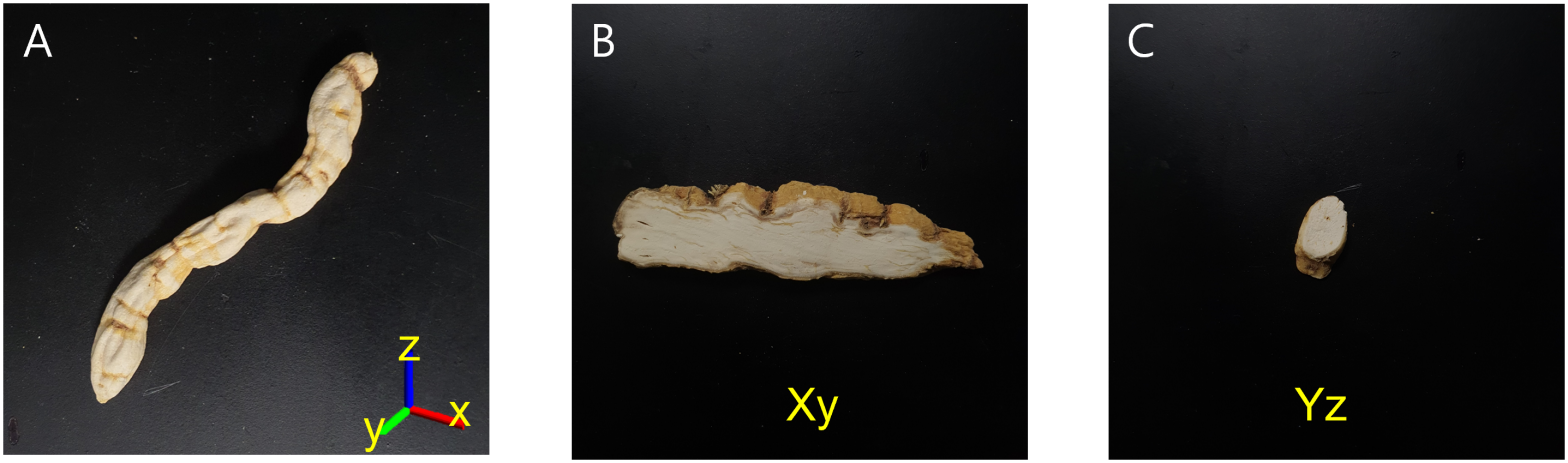
A sample of the root zone of *C. wilfordii* **(A)**, horizontal section (Xy) **(B)**, vertical section (Yz) **(C)**.

*C. wilfordii* and *C. auriculatum* belong to the same Asclepiadaceae family and are physically quite similar because they have hypertrophied roots. Tuberous roots, in particular, are generally seen in horizontally or vertically cut forms. The entire length is approximately 7 to 12 cm, and the diameter is approximately 0.5 to 1.5 cm. **Figure 1A** depicts the overall shape of *C. wilfordii* roots. It is commonly supplied on the market in the shape of B and C in **Figure 1**, and samples cut in this manner are difficult to match with *C. auriculatum*. **Figure 1B** exhibits the X-Y axis with the long side of the root as the reference axis, while **Figure 1C** illustrates the Y-Z axis. The truncated B and C forms of *C. wilfordii* and *C. auriculatum* are to be recognized individually in this study. As a result, two cut sections were photographed, yielding 800 images, 200 of each of the four classes.

**Figure 2:**
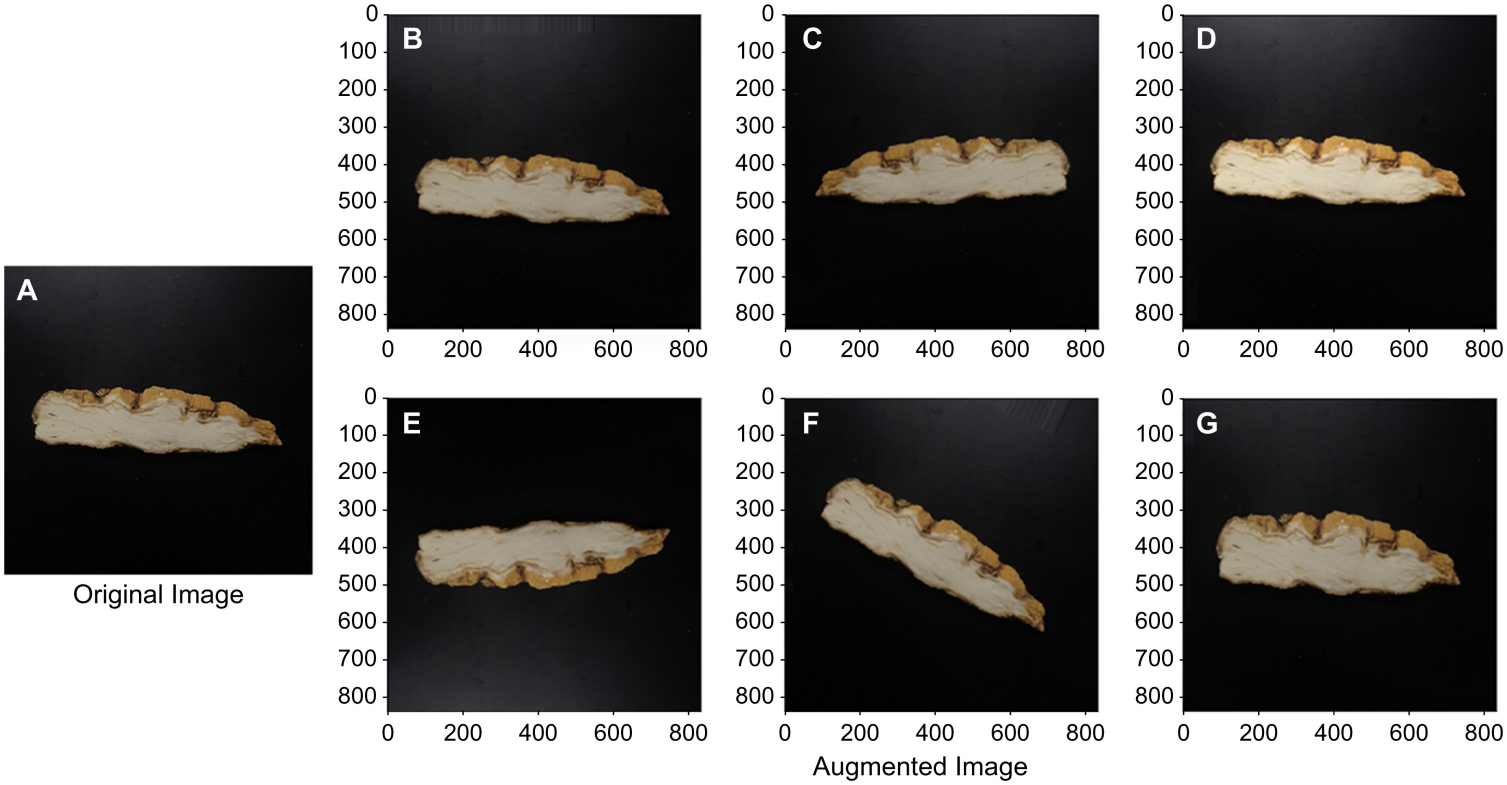
Original image of the horizontal section sample of *C. wilfordii* **(A)** and augmented images **(B)** Vertical shift; **(C)** Horizontal Flip; **(D)** Brightness; **(E)** Vertical Flip; **(F)** Rotation; **(G)** Zooming.

The amount of training data is significant for constructing a model in AI learning. The data augmentation method is primarily used when obtaining a large number of image data is difficult. Data augmentation was done on the captured images in this study using the generally used image augmentation approach depicted in **Figure 2** below. In **Figure 2**, A is the original image, and B-G are images augmented using a commonly used image processing technique.

In this study, 100 images were applied to each augmentation method by selecting original images using a random function to avoid overfitting through a specific technique in the six preprocessing techniques. In the following, we investigate how the learning and verification performance is affected by whether or not image augmentation is conducted for the dataset before applying it to the AI model.

### 2.2 Development of CNN classification model

#### 2.2.1 Applied CNN model

**Figure 3:**
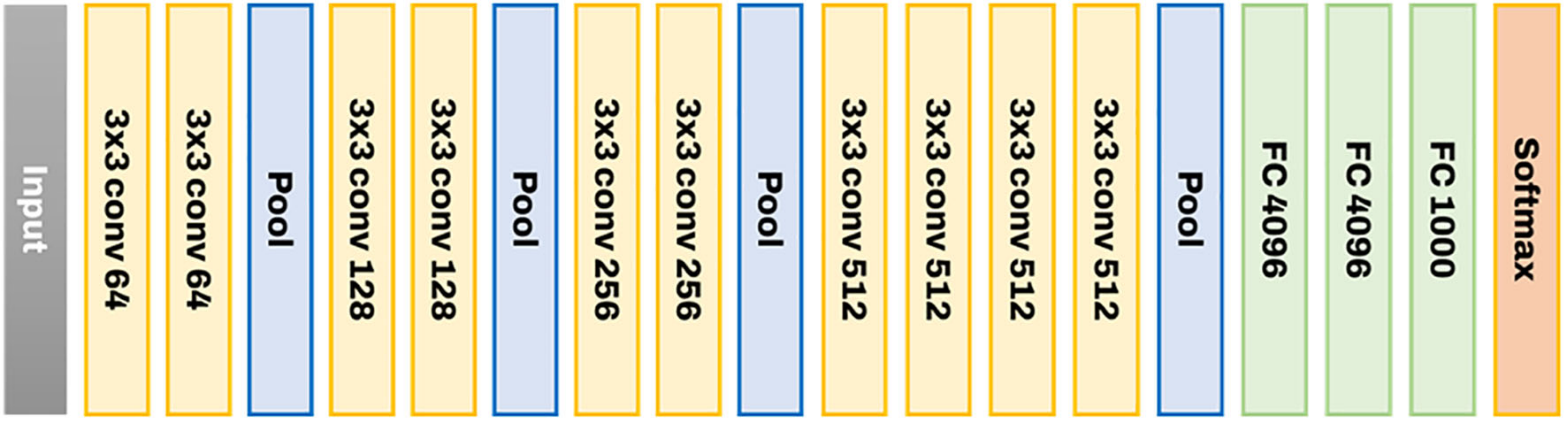
The structure of VGGnet-19 used to develop the image classification model.

In RGB image object recognition and classification, the 2D CNN model outperforms existing image processing approaches (Rawat and Wang, 2017). Two models with good performance and applications in various industries were chosen from among the most frequently reported model structures in 2D image categorization and utilized as comparison groups in this study. Moreover, the same inputs and outputs were employed as in the previous 2D CNN proposal. VGGnet-19 is the first 2D-CNN algorithm identified for comparison. VGGNet is a model proposed by Simonyan and Zisserman (2014) in the 2014 ILSVRC, which came in second place after GoogLeNet. However, it is used more often since it is structurally simpler than GoogLeNet, thus, easier to understand and test. **Figure 3** illustrates the structure of VGGnet-19. Layers are learned from the input on the left to the softmax on the right of the diagram. In general, it consists of a conv layer, which serves as the core of the CNN structure, and a pooling layer, which reduces data space. Before categorization, the process’s ultimate outcome is flattened one-dimensionally through a fully linked layer. The softmax function is then used for categorization. In the case of VGGnet, the filter of the conv layer was set to 3 × 3 with stride = 1, and the ReLU function was utilized as the activation function.

**Figure 4:**
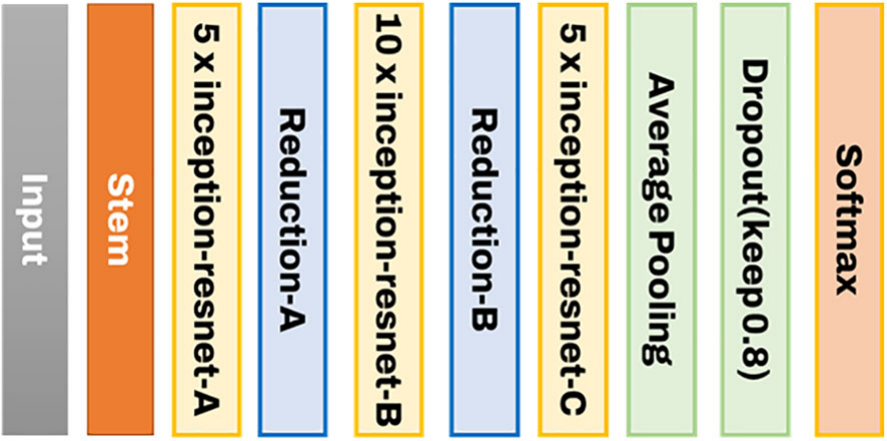
The structure of Inception-ResNet used to develop the image classification model.

Inception-ResNet, proposed by Szegedy et al. (2017), is the second CNN classification model used. Inception-ResNet was designed to successfully broaden and deepen the Inception neural network. Furthermore, better learning speed was reported by adopting a simpler and more uniform structure than Inception-v4 and more Inception modules. **Figure 4** depicts the entire structure of Inception-ResNet.

**Figure 5:**
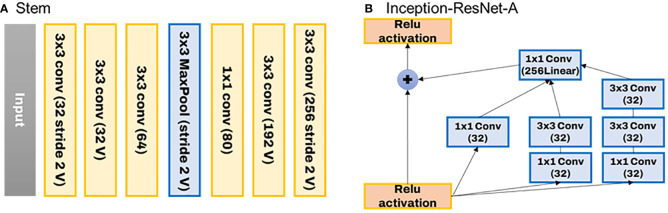
Stem structure used in Inception-ResNet **(A)** and ResNet-A layer of the residual connection structure **(B)**.

The structure of the stem used has a conv-net structure commonly seen in general CNN structures, as shown in **Figure 5A** below. This is because the inception effect is not significant at the beginning of the layer. The biggest feature of Inception CNN is that the matrix operation itself is processed densely while connecting the Conv layer sparsely. As shown in **Figure 5B**, a residual connection structure is configured to implement a function that calculates by combining the result of the previous layer with the result of the current layer. This has significantly affected the improving learning speed. The Resnet-A layer has a 35 x 35 grid, and the Resnet-B layer has a 17 x 17 grid. The modules to reduce this are consecutively composed of Reduction-A and B.

### 2.3 Local interpretable model-agnostic explanation for XAI

LIME is a method that specifically implements the local surrogate model. Surrogate models learn to “approximate” the predictions of the underlying black box model. However, we focus on learning a local surrogate model to explain individual predictions rather than learning a global surrogate model. A good approach to using LIME is to forget about the original dataset and see why the model returned a particular result. Therefore, LIME tests what changes occur to model predictions when the input data is modified and creates a new dataset consisting of perturbed samples and corresponding predicted values of the black box model. Based on this, an interpretable model weighted by the proximity of the sampled observation to the observation (instance) of interest is learned. A local surrogate model has interpretability constraints can be expressed as follows (Ribeiro et al., 2016):


…[1] $$explanation\left(x\right)=\;argmin{\;}_{g\in G}\;L\left(f,g,\pi_{x}\right)+\Omega \left(g\right)$$

Here g denotes a model that can explain the observed value x and minimizes the loss function L. While keeping the complexity of the model Ω(g) lower, the loss measures how close the predictions of the explanatory model are to those of the original model f. The loss function L here is expressed as follows (Ribeiro et al., 2016):


…[2] $$L\left(f,g,\pi_{x}\right)=\;\sum_{z,z^{\prime }\in \;Z}\pi_{x}\left(z\right){\left(f\left(z\right)-g\left(z^{\prime }\right)\right)}^{2}$$

Here G is a set of possible explanations, including a general linear regression model, and $\pi_{x}$ is the measure of proximity indicating how close to the observed value (x) that we consider for the explanation.

**Figure 6:**
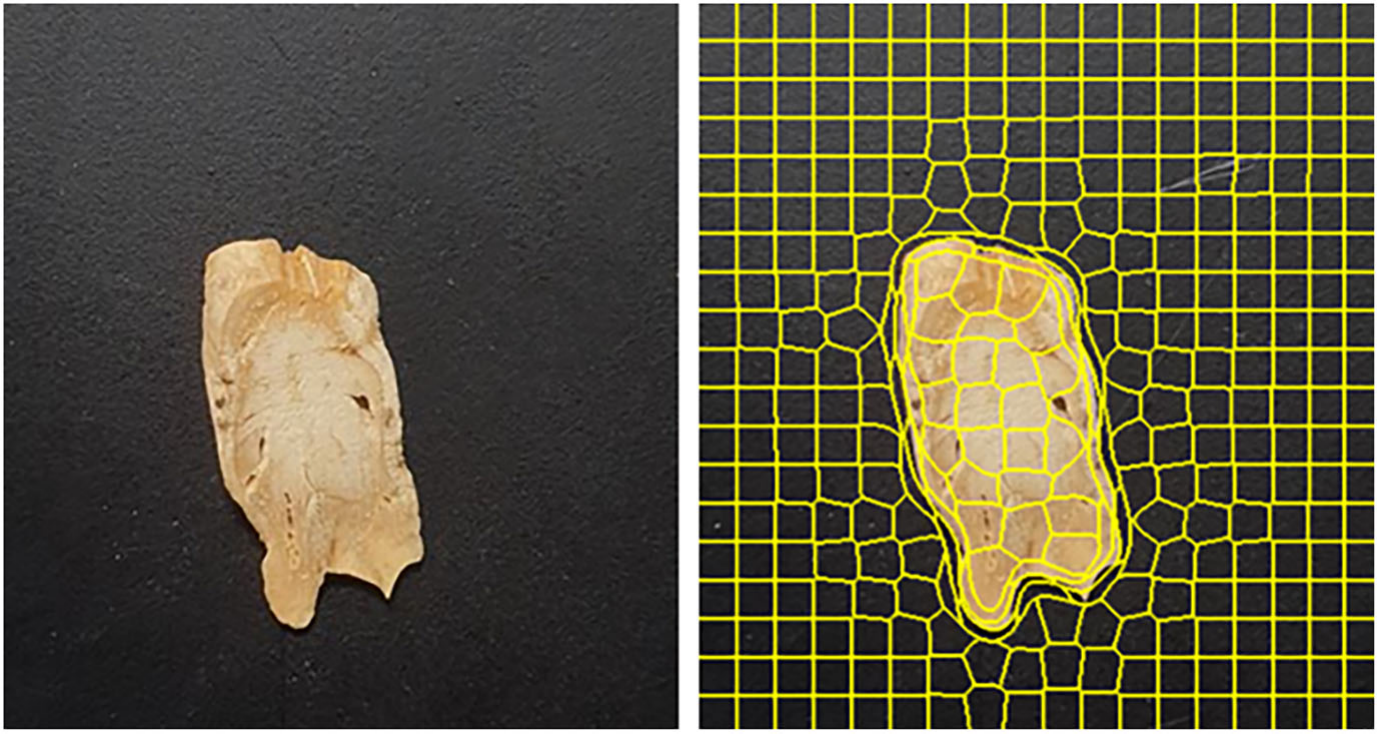
Top view of the cut *C. wilfordii* (right) and image segmented by the SLIC algorithm for superpixels (left).

Images’ data format is a cluster of pixels, and it is difficult to determine how significantly a single pixel contributes to class determination. Individual pixel changes have little effect on the model’s predictions, and image transformation is accomplished through the superpixel segmentation and masking procedure. Superpixel is a method for image classification that collects and groups perceptually important pixels (Wang et al., 2017). Adjacent pixels with comparable qualities (e.g., color, brightness) are clustered together to form a big pixel or superpixel. It signifies that the image will be processed in units of superpixels rather than units of pixels. When we look at an image, we look at related parts together rather than each pixel separately. As a result, it can be demonstrated that processing images in superpixel units is a more natural (human-like) way (Achanta et al., 2012). Thus, superpixel and XAI technology were coupled to provide an auxiliary metric for sensory evaluation. The image was segmented using Simple linear iterative clustering (SLIC), one of the superpixel segmentation algorithms. SLIC is an algorithm applying k-means for superpixel generation and has two key differences. 1) Reduce the number of distance computations in the optimization by limiting the search space to an area equivalent to the superpixel size. This becomes linear in the number of pixels N and can lower the complexity irrespective of the number of superpixels. 2) A weighted distance measurement maintains consistency while using color and geographical proximity. In this image classification and XAI application, around 250 pieces were processed using the SLIC algorithm on 512 × 512 video images (**Figure 6**).

### 2.4 Model performance evaluation method

The developed model is a classification model for differentiating *C. wilfordii* and *C. auriculatum*, and its performance was evaluated based on accuracy, F1 score, and the receiver operating characteristic (ROC) curve. Accuracy is a metric that takes into account the situation in which the model infers two classification labels and predicts true as true and false as false, and is expressed as follows:


…[3] $$Accuracy=\;\frac{TP+TN}{TP+FN+FP+TN}$$

True Positive (TP): Predict the answer that is actually true as true (correct answer)

False Positive (FP): Predict the answer that is actually false as true (wrong answer)

False Negative (FN): Predict the answer that is actually true as false (wrong answer)

True Negative (TN): Predict the answer that is actually false as false (correct answer)

The F1 score is one of the statistics that define the classification accuracy and recall rate, which are combined into a single statistic. Here, the harmonic average was determined, not the standard average. Its purpose is to ensure that the F1 score has a similar low value as precision and recall, which are close to 0. The equation for the F1 score is as follows:


…[4] $$F_{1}=2\;\cdot \;\frac{1}{\frac{1}{recall}+\frac{1}{precision}}=2\;\cdot \;\frac{precisioin\;\cdot recall}{precision+recall}$$

**Figure 7:**
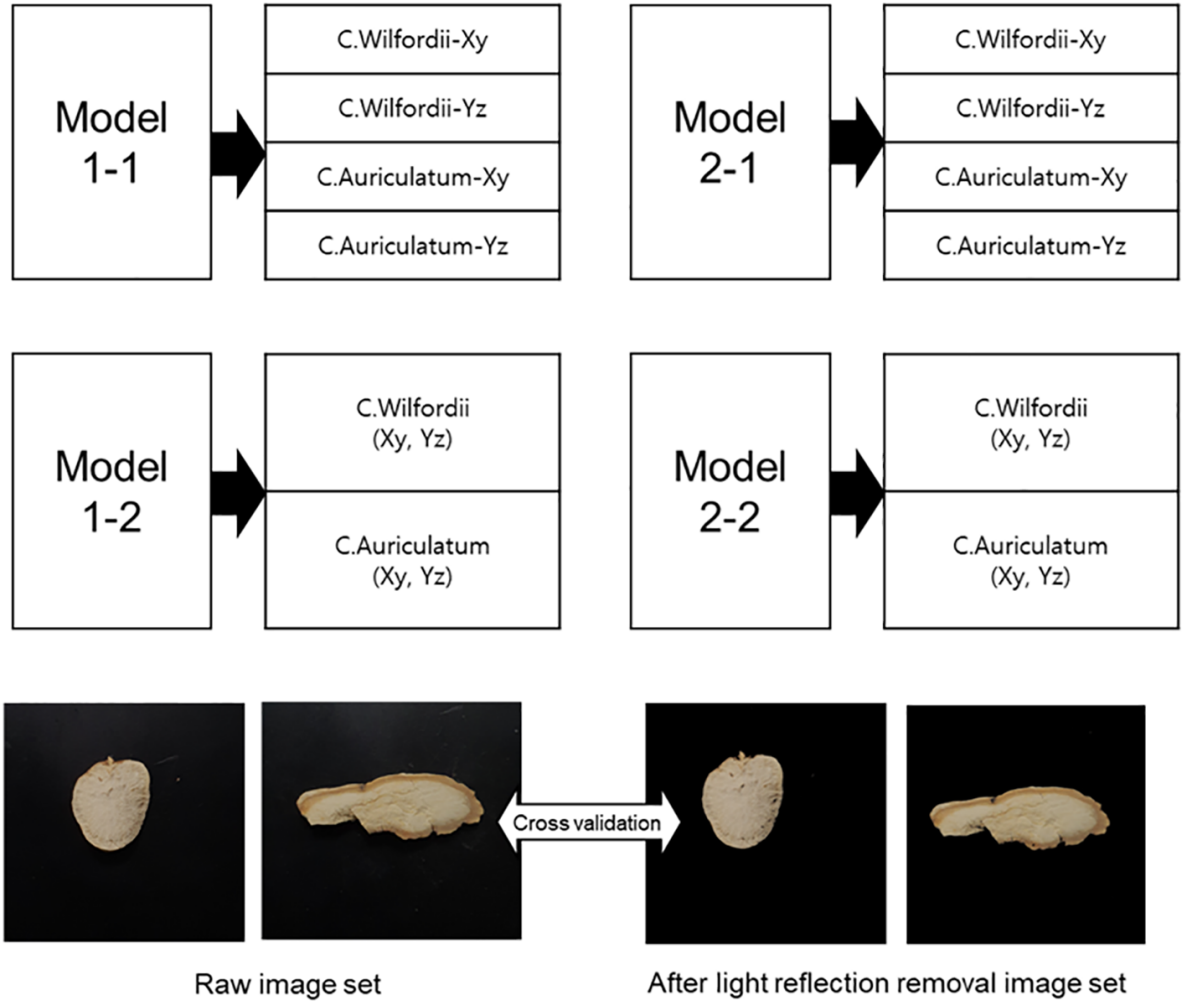
Training data groups to compare the performance of CNN models applied to develop the *C. wilfordii* and *C. auriculatum* classification models.

**Figure 6** depicts the classification of the training data set used to compare the performance of the CNN model used to create the *C. wilfordii* and *C. auriculatum* classification models. Models 1-1 and 1-2 apply unprocessed video images without a separate light reduction procedure. Models 2-1 and 2-2, which will be contrasted with this, eliminate background light reflection. Except for the sample area, the background was transformed to picture RGB (0,0,0) or black. The classification goals of Model 1-1 and Model 1-2 were assigned distinct labels to classify the two shortened forms of *C. wilfordii* and *C. auriculatum*. Models 1-2 and 2-2 defined both portions as a single medicinal product label and then classified each variant (**Figure 7**). Moreover, whether a region that could serve as a sensory metric could be detected was determined by applying LIME to each model.

The CNN Inception-ResNet and VGGnet models were constructed using the Tensorflow and Keras libraries in Python 3.8. The GPU used to calculate the model was NVIDIA RTX 3090.

## 3 Results

### 3.1 Comparison of performance between CNN Inception-ResNet and VGGnet

**Figure 8:**
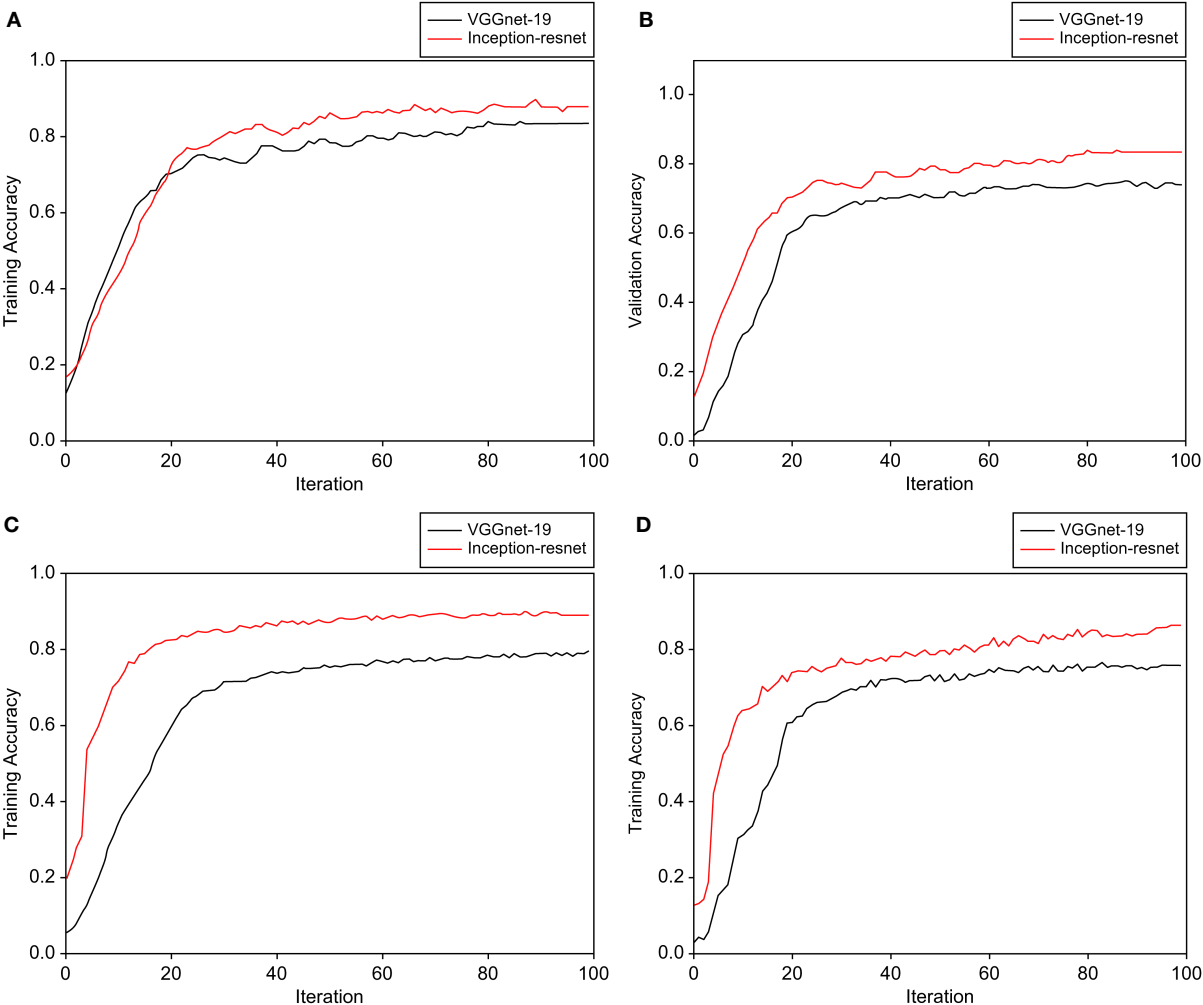
Comparison of the performance of the CNN model applied to develop a classification model for *C. wilfordii* and *C. auriculatum*: **(A)** Classification result of raw image learning model, **(B)** Verification result of the developed raw image-learning model, **(C)** Classification result of the learning model after light removal treatment, and **(D)** Verification result of the developed light-cancellation image set model.

**Figure 9:**
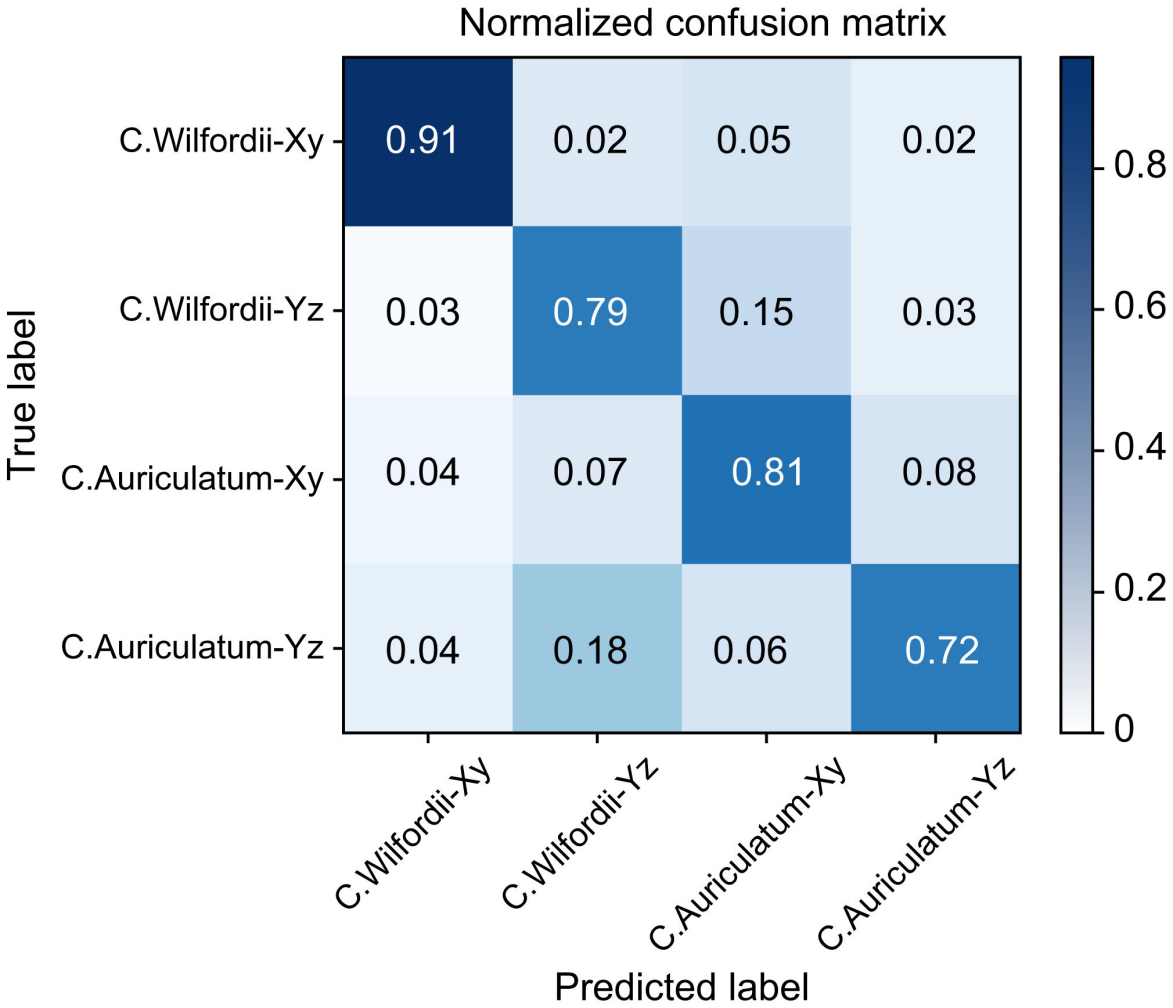
Four-label classification confusion matrix of the CNN Inception-ResNet.

The collected images of *C. wilfordii* and *C. auriculatum* were divided into four classes based on the cut section, and learning and verification were performed. Furthermore, the learning and verification performances were compared using two CNN models. Through the augmentation of the original 800 images, around 3200 images were utilized. The learning-to-verification ratio was 8:2, and the data was split. **Figure 8** compares the outcomes of data models 1-1 and 2-1. In the performance of the two CNN models, the CNN Inception-ResNet structure clearly exhibited faster learning speed and higher classification accuracy than VGGnet-19. The learning accuracy of Model 1-1’s Inception-ResNet was 0.835, while its verification accuracy was 0.812. The learning accuracy of VGGnet was 0.776, while the validation accuracy was 0.710. The classification accuracy of model 2-1 with light reflection removal was 0.821 at 0.861 verification accuracy in Inception-ResNet training and 0.701 at 0.712 verification accuracy in VGGnet training. **Figure 9** is a confusion matrix displaying the classification accuracy of Inception-four ResNet’s classes. The average separation accuracy of the xy-cut sections in collected samples of *C. wilfordii* and *C. auriculatum* was 0.86, but the yz-cut sections had a lower accuracy of 0.76.

With the implementation of the data augmentation method, the accuracy in the test set showed a noteworthy improvement of 0.1 to 0.15. This indicates that there was a significant boost in learning performance, even with a limited number of samples, while also mitigating the potential risk of overfitting.

### 3.2 Result of removing light reflection of an image through the application of LIME

Models 2-1 and 2-2 corroborated the model classification outcome by specifying two cut sections as one class. **Table 1** summarizes the results. Models 1-1 and 1-2, which split and classified cut sections into classes, showed higher classification performance in general. In particular, the accuracy of VGGnet-19 increased considerably from 0.71 to 0.80. The performance of Inception-ResNet improved slightly as well, and the validation set classification accuracy in Model 2-2 was around 0.862.

**Table 1:** Validation set accuracy and F1 scores of classification cases 2-1 and 2-2.

	Inception-ResNet		VGGnet-19	
	Accuracy	F1 score	Accuracy	F1 score
Model 1-2	0.855	0.834	0.811	0.806
Model 2-2	0.862	0.851	0.805	0.801

**Table 2** summarizes the performance cross-validation results of Model 1-2, a classification model developed using raw images, and Model 2-2, which was developed using images with light removal processing to extract sample regions. The performance of the model trained with raw images was confirmed to be much lower when categorizing images with light reflection removed. The accuracies of 0.641 and 0.671 were validated as the performance of the two CNN models, which failed to show significant performance in classifying the two classes.

**Table 2:** Cross-validation accuracy and F1 score results of classification cases 2-1 and 2-2 based on the developed CNN model.

	Inception-ResNet		VGGnet-19	
	Accuracy	F1 score	Accuracy	F1 score
Model 1-2 to Model 2-2	0.641	0.560	0.671	0.622
Model 2-2 to Model 1-2	0.821	0.798	0.742	0.712

**Figure 10:**
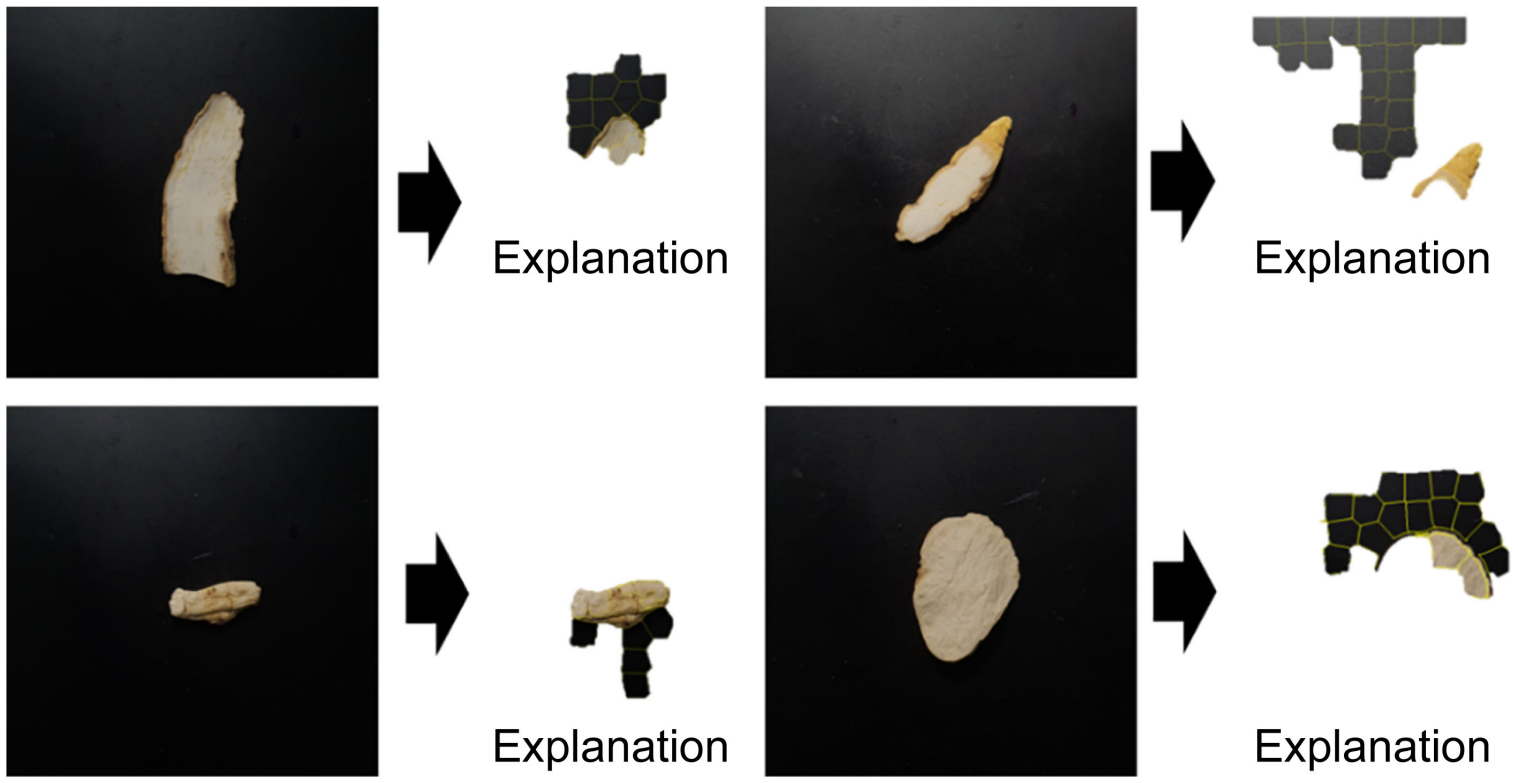
Result of finding explanation parts through LIME analysis for the Inception-ResNet model trained by raw images.

**Figure 11:**
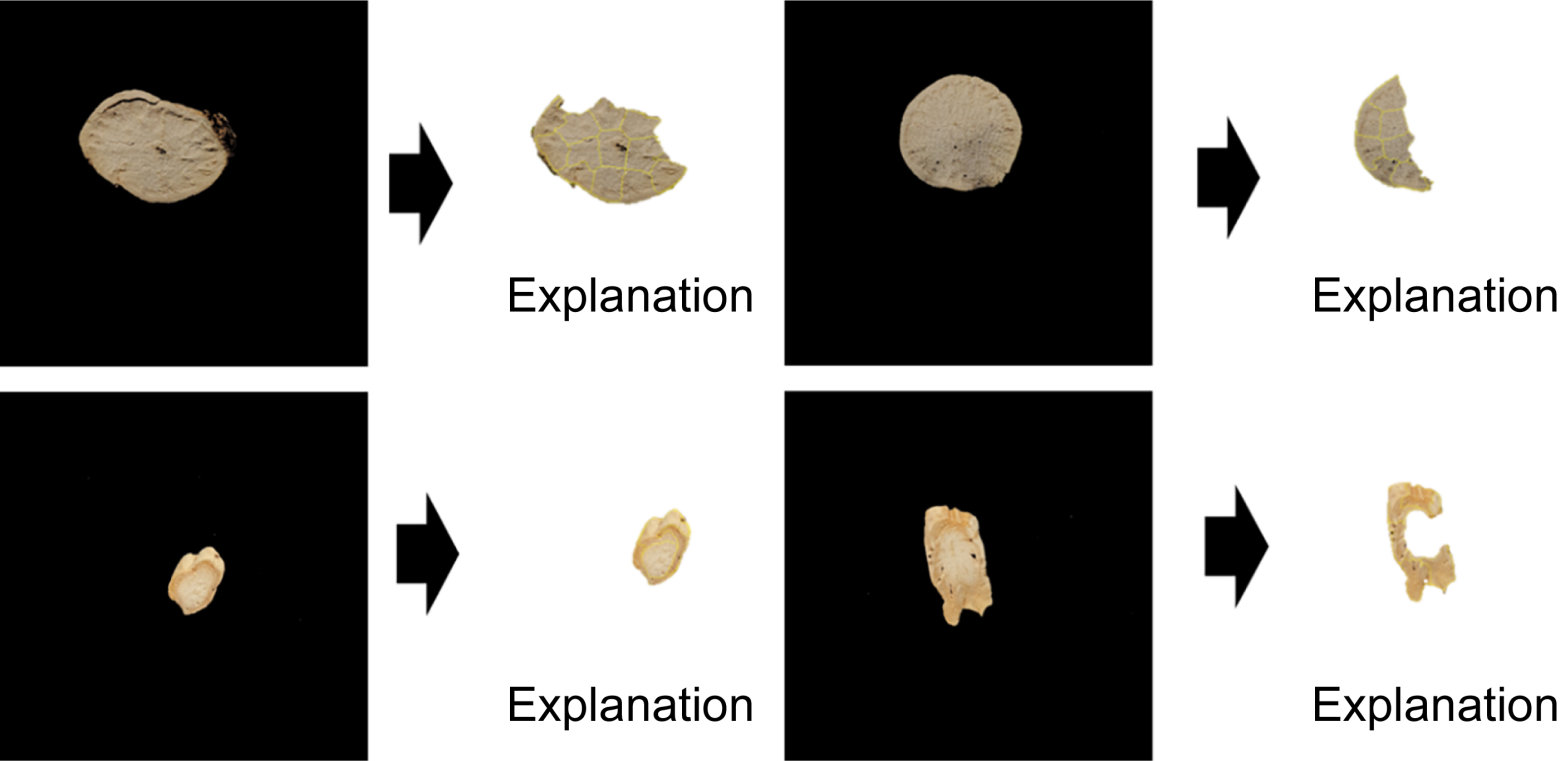
Result of finding the explanation part of the Inception-ResNet model through LIME analysis for the Inception-ResNet model trained by images from which the reflected light on the background has been removed.

These results validated the local active area identified by the LIME algorithm as the cause. **Figures 10**, **11** depict the trained model as a raw image and the image with the background’s reflected light removed, respectively. They display the outcome of discovering an explanation component through LIME analysis of the trained model. **Figure 10** depicts not only the cross-section of the sample but also the descriptive local area inside the region of reflected light in the backdrop. Conversely, **Figure 11** confirmed the outcome of locating the explanatory component in the sample area.

## 4 Discussion

Medicinal plants have similar species that can be readily jumbled in distribution, and distinguishing them requires a very high level of sensory evaluation experience. The medicinal plant species *C. wilfordii* and *C. auriculatum* exhibit similar morphologies, and they are commonly intermingled in the distribution process. These kinds are exceedingly tough to find professionals that can recognize them in the field. As a result, disruptive molecular analysis, such as physicochemical component analysis or RNA analysis, can provide confidence in their categorization accuracy (Han et al., 2016). However, this study demonstrated that it is possible to differentiate between *C. wilfordii* and *C. auriculatum* efficiently using AI image classification technology, which is rapidly evolving.

In particular, the architectures of CNN models Inception-ResNet and VGGnet-19 were used for classification. Inception-ResNet demonstrated improved results in terms of performance and learning rate. Regarding two algorithms, the average processing speed for the same training data (approximately 2400 images) in a single iteration was 12.45 ± 1.25s and 26.22 ± 2.76s, respectively, based on the GPU specifications used. A strong classification performance of around 0.862 was confirmed in the validation set. The two medications are mostly available in sliced form. When 4 classes were identified through 2 representative cross-sectional examples, the highest verification result was roughly 0.835. The xy-axis truncated shape demonstrated higher classification accuracy. In fact, the model appears to have limitations if the model is inferred or given in a crushed or another form while the sample is not cut. In this study, images were captured at a consistent height and position of the sample in the top view, where the cross section is most visible. Thus, the classification performance loss is to be expected when testing the classification performance of images from multiple camera angles. This appears to be solvable by acquiring more diversified and numerous images and applying them for training.

LIME, an AI explainability technique, was employed in this study to investigate whether it may be used as an auxiliary metric for sensory evaluation by marking the explanatory component among cross-sections of categorized medicinal materials. **Figure 11** confirmed that the technique was explanatory in the sample’s local area and suitably adaptable. Light reflection on the background is evident to the naked eye in the raw image data of the collected image. LIME yielded sample results with an explanatory ability to model training in this domain. This appears to be due to the model detecting the impact of the surrounding environment during image collecting; this is expected to be validated later through LIME analysis of several images taken in different situations. Only the sample had model explainability in the area where the background light was deleted. It was also established that the surface of the sample, rather than the center, is the area primarily stimulated between the *C. wilfordii* and *C. auriculatum* samples; this can also be perceived as the overall shape of the cross-sectional contour.

Explainable AI refers to the technology that provides interpretable forms of the prediction results generated by machine learning models. This technology helps to understand and analyze the prediction results of the model, making the model’s prediction results more reliable. The image recognition and classification technology in video and images has already been extensively researched, but it is difficult to judge whether the learning intentions of the model, which is developed as a black box, match. By utilizing explainable AI technology, it becomes possible to understand how a machine learning model makes predictions, enabling the judgment of whether the model’s learning intentions are consistent. Furthermore, the classification technology of C. wilfordii and C. auriculatum could be used as an auxiliary means for determining herbal medicine in the distribution field if it is connected to a web platform, and it is highly likely that it can be used as a means of providing accurate information to customers by using devices that can acquire images, such as smartphones. This technology can increase the reliability of herbal medicine sales and help customers use herbal medicine correctly.

## 5 Conclusions

Images were collected in this work to categorize *C. wilfordii* and *C. auriculatum*, which were then processed and put into a deep-learning classification model to corroborate the results. For image classification, the architectures of Inception-ResNet and VGGnet-19 among CNN models were employed for classification. Inception-ResNet demonstrated improved results in terms of performance and learning rate. The validation set confirmed a strong classification performance of around 0.862. LIME was also used to add explanatory characteristics to the deep-learning model. In both situations, the appropriateness of the LIME area was determined using cross-validation. As a result, the raw image was confirmed to be activated in the light reflection area in the surrounding background. When eliminated, the created model’s accuracy declined dramatically from 0.855 to 0.641. The second case model designed to choose the sample region, on the other hand, maintained an accuracy of 0.8 or higher even after cross-validation. This Explainable AI has the potential to be employed as an auxiliary metric in the sensory evaluation of therapeutic compounds in future.

## Data availability statement

The raw data supporting the conclusions of this article will be made available by the authors, without undue reservation.

## Author contributions

Conceptualization, D-HJ and SP. Methodology, D-HJ. Validation, H-YK and JW. Formal analysis, JW. Data curation, D-HJ. Writing—original draft preparation, D-HJ. Writing—review and editing H-YK and JW, Visualization, SP. Supervision, D-HJ. All authors contributed to the article and approved the submitted version.
